# Bilateral multifocal and recurrent chorioretinopathy - case report

**DOI:** 10.22336/rjo.2022.67

**Published:** 2022

**Authors:** Andreea-Petra Cristea, Cristina Stan

**Affiliations:** * Department of Ophthalmology, Emergency County Hospital, Cluj-Napoca, Romania; ** Department of Ophthalmology “Iuliu Hațieganu” University of Medicine and Pharmacy Cluj-Napoca, Cluj-Napoca, Romania

**Keywords:** multifocal chorioretinopathy, subretinal fluid, retinal pigment epithelium

## Abstract

**Objective:** The aim of this report is to highlight a rare condition that raises serious diagnosis and treatment difficulties.

**Case presentation:** A 34-year-old male patient presented at the Department of Ophthalmology accusing reduced visual acuity (VA), dyschromatopsia and slight photophobia in his left eye (OS). Posterior pole examination revealed serous retinal detachment superior to the optic nerve head in his right eye (OD) and a well-defined macular oedema in the OS. Optical coherence tomography (OCT) confirmed the presence of subretinal fluid accumulations, fundus fluorescein angiography (FFA) revealed punctate hyperfluorescent pinpoint foci in the macular region of both eyes in the early venous phase and dye pooling in the late phase. The first diagnosis was Probable Vogt-Koyanagi-Harada (VKH) syndrome, but the evolution under corticosteroid therapy and shifting of the position of the serous retinal detachments in time, changed the diagnosis to multifocal, recurrent central serous choroidopathy. The patient received treatment with anti-vascular endothelial growth factor (VEGF) agents and presented multiple episodes of partial remission and shift of the subretinal fluid.

**Conclusions:** The persistent, recurrent, multifocal and bilateral exudative retinal detachments raised significant diagnosis difficulties. In the absence of a well-established treatment, the current prognosis is unfavorable.

**Abbreviations:** MARC = multifocal and recurrent choroidopathy, CSCR = Central Serous Chorioretinopathy, RPE = retinal pigment epithelium, CFH = complement factor H, VA = visual acuity, OD = right eye, OS = left eye, OCT = ocular coherence tomography, VEGF = vascular endothelial growth factor, FFA = Fundus fluorescein angiography, p-ANCA = Perinuclear anti-neutrophil cytoplasmic antibodies, PR3 = IgG antibodies against proteinase 3, ANA = antinuclear antibodies, CIC = Circulating immune complexes, CMV = Cytomegalovirus, VKH = Vogt-Koyanagi-Harada.

## Introduction

Multifocal, recurrent serous chorioretinopathy is a rare pathology first described as an independent entity known as MARC (multifocal and recurrent choroidopathy) syndrome in 1984 [**1**]. Nowadays, it is considered the rare chronical form of Central Serous Chorioretinopathy (CSCR) affecting most frequently otherwise healthy middle-aged men with Asian descendants. Most of the time, both eyes are affected with an asymmetric pattern of the neurosensory retinal detachments and RPE changes determining reduced visual acuity, blurry vision and dyschromatopsia. The pathogenesis and risk factors of this disease are not yet fully understood, but the most investigated element leading to the chorioretinopathy is the exposure to abnormal cortisol levels [**2**-**5**]. Another highly investigated risk factor for the aetiology of multifocal central serous chorioretinopathy is the exposure to significant stress levels [**2**]. Studies confirm that the first manifestations of the disease are highly linked to major life events and emotional discomfort occurring around a year prior to the initial symptoms [**6**]. A strong corellation was identified between multifocal serous chorioretinopathy and type A personality characterized by perseverance, competitivity, in some cases leading to aggressiveness and hostility [**2**,**3**]. Patient suffering from the chronic form of CSCR tend to develop gastroesophageal reflux disease and peptic ulcer more frequently than the general population, the sympathetic overactivation being the most likely explanation [**3**].

Generally, the classical form of CSCR is a sporadic, self-limiting disease, though there have been case reports of rare chronical forms affecting multiple members of the same family. Up until now, an intrafamilial pattern of transmission has not been identified, but there is significant interest in identifying a genetic component for this pathology [**7**,**8**]. A study published by de Schubert et al. in 2014 found that the *CDH5*, a gene responsible for the synthesis of an adhesion glycoprotein found at the choroidal capillaries’ endothelial level and suppressed by the exposure to glucocorticoids, may play a significant role in the pathogenesis of Central Serous Chorioretinopathy [**7**]. Miki et al. also found a possible relationship between the CFH (complement factor H) polymorphisms and CSRC via adrenomedullin [**8**].

## Case Report

The current study presents the case of a 34-year-old male patient with a rare form of bilateral multifocal recurrent serous chorioretinopathy.

**Fig. 1:**
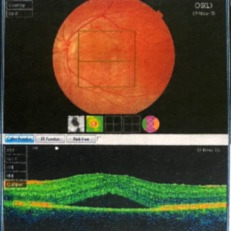
Optical Coherence Tomography. Horizontal macular section of the OS at the beginning of the symptoms: accumulation of sub-retinal fluid

The first manifestations of the disease occurred 3 years prior to the initial presentation in our department, when the reduced visual acuity (VA) and blurry central vision in his left eye (OS) led the patient to a private ophthalmology clinic. At that time, his visual acuity measured with the Snellen chart was 1 and 0,9 in his in his right (OD) and left eye, respectively. The posterior pole examination revealed serous macular detachment in his OS with ocular coherence tomography (OCT) confirming the accumulation of sub-retinal fluid leading to the diagnosis of central serous chorioretinopathy (**Fig. 1**). The patient was held under observation for 3 months and, due to lack of remission, the ophthalmologist decided to perform intra-ocular injection with an anti-vascular endothelial growth factor (VEGF) agent into the affected eye. 

**Fig. 2:**
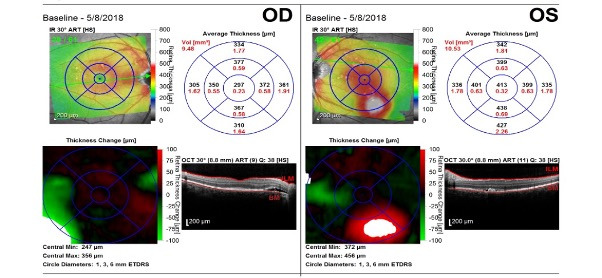
Optical Coherence Tomography. Horizontal macular section: OD: subtle subretinal fluid accumulations in the interpapilomacular area OS: significant subretinal fluid deposit inferotemporal to the fovea

**Fig. 3:**
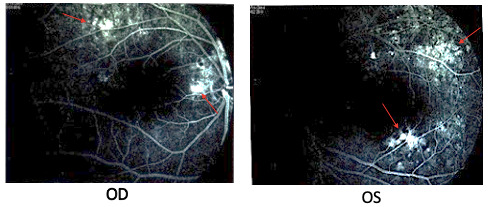
Fundus fluorescein angiography. Late phase: Fluorescein pooling

In the absence of significant ocular manifestations, the patient did not perform routine check-ups and presented at the Department of Ophthalmology of the County Hospital years later accusing reduced VA, dyschromatopsia and slight photophobia in his left eye. At that time, the best corrected visual acuity with the Snellen chart was 1 in the right eye and 0,5 in his left one. Posterior pole examination revealed a serous retinal detachment area superior to the optic nerve head in the OD and a well-defined serous macular detachment in the OS. The patient was admitted for further investigations. The OCT examination confirmed subtle subretinal fluid accumulations in the interpapilomacular area of the right eye along with a significant subretinal fluid deposit inferotemporal to the fovea of the left one (**Fig. 2**). Fundus fluorescein angiography (FFA) identified punctate hyperfluorescent pinpoint foci in the macular region of both eyes, in the early venous phase, and dye pooling surrounding the optic disc in the OD, and superior and inferior to the fovea in the OS, in the late phase. A surprising element was the lack of typical leakage spots on FFA, making laser photocoagulation treatment impossible (**Fig. 3**).

Various blood tests were performed in order to exclude a possible common autoimmune or infectious disease as being responsible for the chorioretinal changes. Complete blood count, perinuclear anti-neutrophil cytoplasmic antibodies (p-ANCA), IgG antibodies against proteinase 3 (PR3), antinuclear antibodies (ANA), circulating immune complexes (CICs) were all within normal limits, *Borrelia* antibodies, *Toxoplasma* antibodies and HIV testing all came out negative. The only positive examination was the Cytomegalovirus (CMV) IgG antibody. Taking into consideration the fact that the patient is an immunocompetent individual, this finding was most likely an incidental one and not the cause of the retinal abnormalities. 

**Fig. 4:**
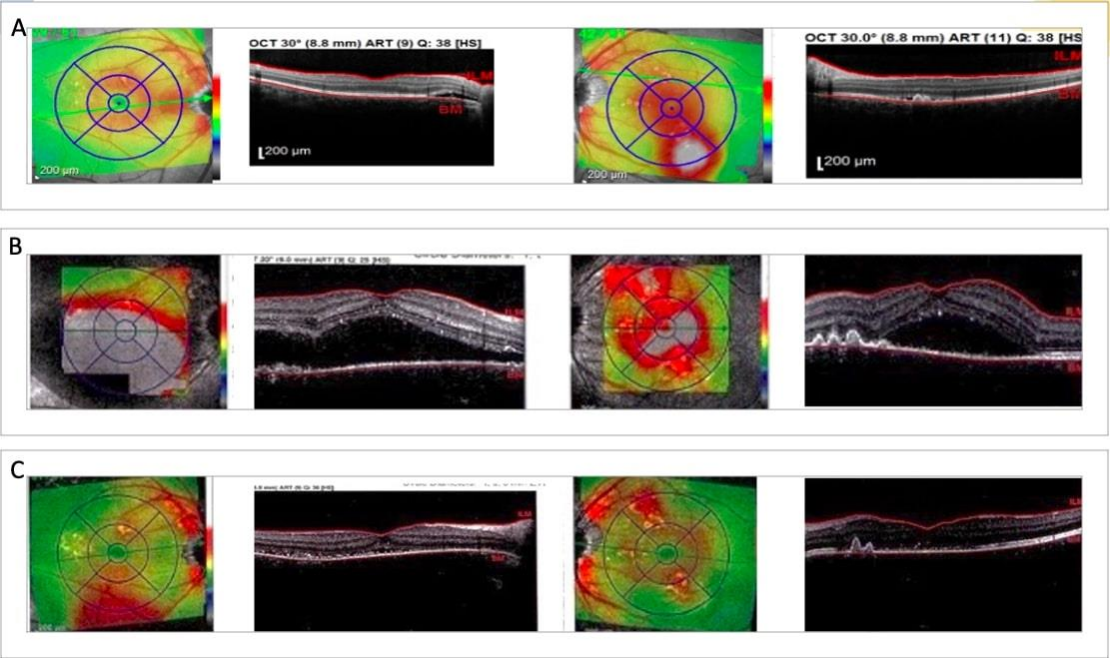
Ocular Coherence Tomography **A.** First examination performed in our clinic **B.** After one month of glucocorticoids treatment: significant macular fluid accumulation with exudative neurosensory retinal detachments **C.** After one month without glucocorticoids treatment: significant fluid regression

Considering the posterior pole changes along with the OCT and FFA findings, we concluded that the most probable diagnosis was Vogt-Koyanagi-Harada syndrome and therefore corticosteroid therapy was initiated. Fundus examination and ocular coherence tomography were performed after one month of treatment and the findings were surprising; instead of the expected fluid regression, the patient’s posterior pole revealed macular elevation with exudative retinal detachments extending temporally in the right eye and significant fluid accumulation at the macular level in the left eye. In the light of current findings, the decision to suppress corticosteroid therapy was made. Two weeks after treatment discontinuation, significant fluid regression from the macular area was observed (**Fig. 4**). To further reduce the exudative retinal detachments and the choroidal vascular hyperpermeability, invitros injections with anti-VEGF agent were also performed with favorable outcome, but without total fluid regression. 

Considering the aggravation of fluid accumulation under corticosteroid therapy alongside the regression after therapy discontinuation, we concluded that the positive diagnosis was not in fact Vogt-Koyanagi-Harada syndrome, but bilateral multifocal, recurrent serous chorioretinopathy. 

The patient is currently kept under close observation by our team with regular check-ups at 3-month interval presenting various posterior pole changes each time with multifocal recurrent serous retinal detachments reduced or shifted spontaneously or under anti-VEGF therapy. 

**Fig. 5:**
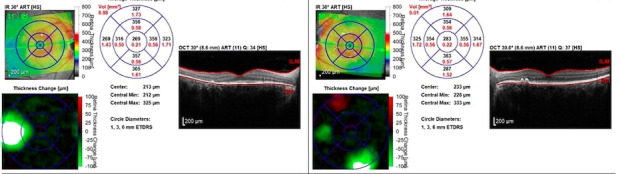
Ocular Coherence Tomography. Most recent evaluation

**Fig. 6:**
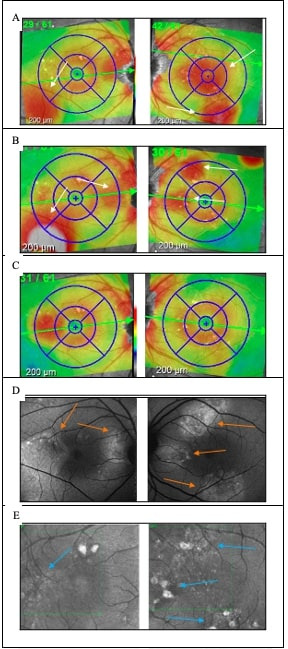
**A, B.** OCT revealing shifting serous retinal detachment areas; **C.** Most recent OCT: former areas of serous detachment being currently attached; **D.** Autofluorescence: multiple hyper-autofluorescencent spots on the site of previous neurosensory retinal detachment areas; **E.** OCT infrared image: hyperreflective dots on the site of previous neurosensory retinal detachment areas (*C, D, E – performed at the same examination)

The most recent posterior pole examination revealed multiple pigmentary lesions disseminated through the retina in both eyes on the site of previous fluid accumulations. OCT revealed no significant serous retinal detachment zones (**Fig. 5**). Autofluorescence was performed identifying multiple hyper-autofluoresencent spots at the site of previous leakage points corresponding to debris at the level of photoreceptors that were previously separated from the RPE (**Fig. 6 D**). The OCT infrared image revealed hyperreflective dots corresponding to the pigmentary changes seen on fundoscopy (**Fig. 6 E**). 

**Fig. 7:**
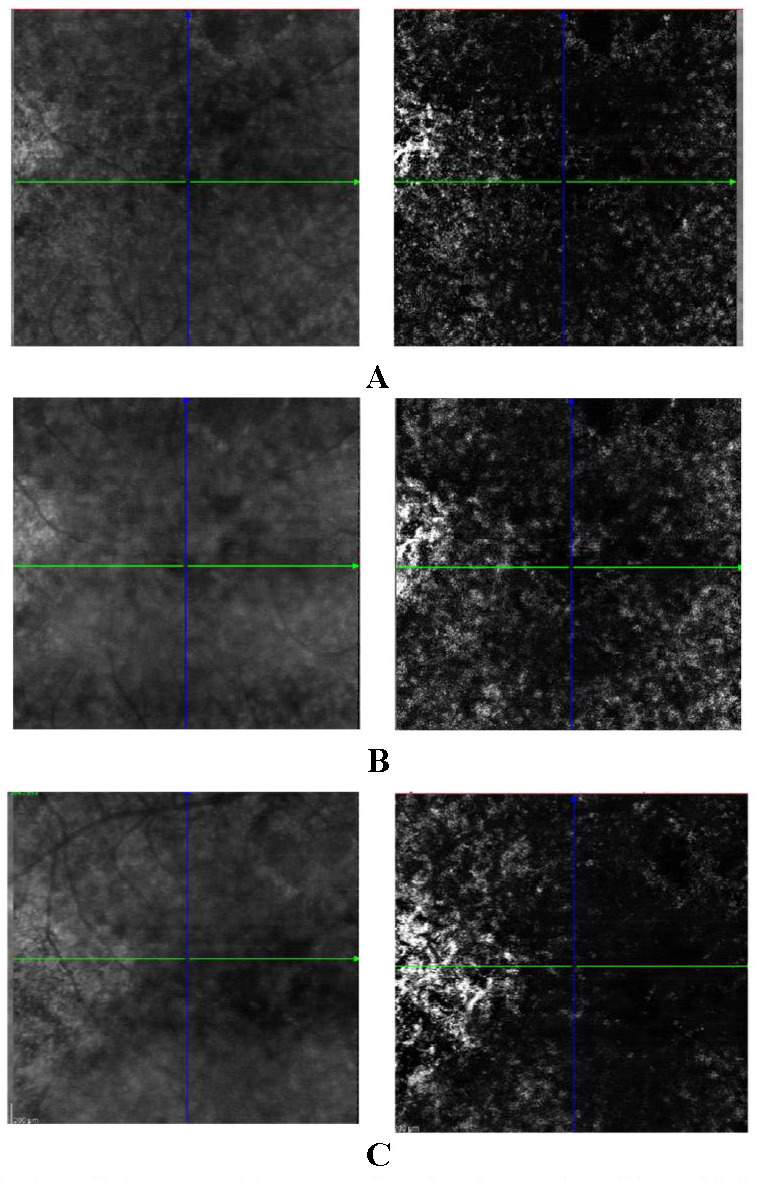
Ocular Coherence Tomography Angiography. Choroidal section. **A. ** Before anti-VEGF injection; **B. ** 3 months after anti-VEGF injection; **C. ** 5 months after anti-VEGF injections

**Fig. 8:**
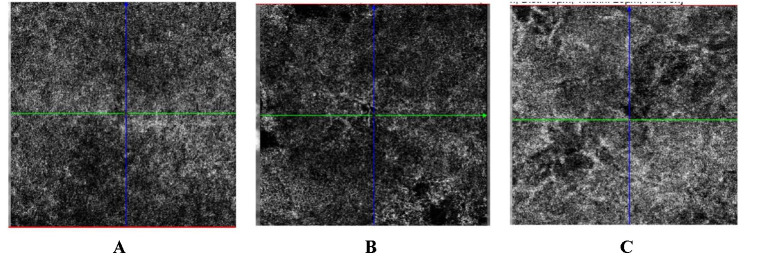
Ocular Coherence Tomography Angiography: choriocapillaris section. **A. ** Healthy age-matched volunteer: normal choriocapillaris; **B. ** Patient with CSCR: areas of decreased signal; **C. ** The patient’s brother: areas of decreased signal

Because choroid neovascularization is an important complication of central serous chorioretinopathy and is associated with a poor prognosis, Ocular Coherence Tomography Angiography was used to assess the disease progression. OCT-A performed 3, respectively 5 months after the last anti-VEGF intravitreal injection revealed a progressively growing new vascular network at the choroid level, thus underlying the need for continuous intraocular treatment (**Fig. 7**). What is more, OCT-A revealed abnormal appearance of the choriocapillaris, the areas with decreased signal could represent either abnormal vessels visibility due to the shadowing effect of the RPE changes, or could be real hypoperfused areas, adding to the theory that central serous chorioretinopathy could result from choroidal vascular autoregulation anomalies (**Fig. 8**). A surprising finding was the discovery of the same choriocapillaris changes on the OCT-A image obtained from the patient’s younger brother with no ocular manifestations and no other OCT abnormalities. This adds more to the hypothesis that the lesions are in facts signs of reduced perfusion due to an underlying choroidal vasculopathy. 

Microperimetry was used to assess the macula function and after the resorption of the serous detachments, normal retinal sensitivity was observed. The patient is kept under close observation with regular check-ups at 3-month interval. 

## Discussion

The current case raised significant diagnostic difficulties, first and foremost due to the multifocal pattern of retinal changes and the recurrent nature of the fluid accumulation. The most important differential diagnoses that our team considered were Vogt-Koyanagi-Harada syndrome, Presumed Ocular Histoplasmosis syndrome, uveal effusion syndrome, and multifocal and recurrent choroidopathy.

VKH is an autoimmune disease in which, in the case of susceptible individuals, a viral infection can lead to the development of antibodies against melanocyte rich tissues such as the eye, inner ear, skin, hair and meninges. It affects most frequently women of color in their 50s, but all genders can develop the pathology. The ophthalmologic diagnostic criteria include the lack of a known ocular pathology or trauma, bilateral posterior pole changes including multifocal serous detachments of the retina or the RPE along with choroid depigmentation. Fundus fluorescein angiography utility is significant, the pathology presenting a characteristic pattern of early spotted hyper fluorescence followed by dye pooling in the subretinal space. Patients suffering from VKH syndrome also present cutaneous, neurological and inner ear manifestations [**9**]. Our patient was referred to the neurology department for a rigorous examination, but no abnormalities were found. The syndrome has a great response to high-dose corticosteroid therapy and our patient’s surprising response led us to disprove this initially possible diagnosis. 

Presumed Ocular Histoplasmosis syndrome is another entity that could be responsible for chorioretinal changes like the ones observed in our patient. Depending on the immune status, patients may present with punctate inner choroidopathy or even panuveitis. The classical ocular triad consists of peripapillary atrophies, macular scars, and choroidal neovascularization. Our patient did not have a history of travelling in the endemic areas, nor presented two of the classic triad components as needed for the diagnosis [**10**,**11**].

Uveal effusion syndrome was also taken into consideration as a possible diagnosis. It represents a recurrent abnormal fluid extravasation from choriocapillaris that can lead to retinal serous detachments and secondary RPE changes. It is considered a diagnosis of exclusion after eliminating more frequent causes of fluid effusion such as trauma, surgery, and scleritis [**12**].

Multifocal, recurrent serous chorioretinopathy was the final diagnosis in our case. The patient’s gender and age corresponded to those of a patient with typical CSRS, but the chronic variant is rare in Caucasian individuals [**1**]. Our patient does not present the classic risk factors: he was not exposed to corticosteroid therapy before admission in the ophthalmology clinic, nor did he declare the presence of a significantly stressful life event prior to the beginning of the ocular symptoms. What is important to mention is the fact that our patient is an entrepreneur and admits to being under constant, but manageable levels of stress. We should consider that subjective reports of emotional discomfort are not the most reliable tools, studies underlining that both perception and description of stress exposure vary significantly from one individual to another, some seeing it as a sign of weakness and therefore denying it [**13**]. As it was previously stated, type A personality is closely linked to central serous chorioretinopathy. We provided our patient with a personality test (Type A Personality Test from www.psychologytoday.com), which revealed the presence of type A specific characteristics such as: perfectionism and competitivity, but as a whole he was in a grey area between type A and B personality. Peptic ulcer is also linked to CSRC, the patient complained of specific symptoms such as: pain in the epigastric region related to meals and heartburn, but never presented to the gastroenterologist for a clear diagnosis. 

Ocular coherence tomography angiography proved to be more sensitive than conventional dye angiography in detecting type 1 choroidal neovascularization in patients with pachychoroid spectrum diseases, including CSRC [**14**]. In our case, the most recent OCT-A performed 5 months after the last anti-VEGF injection revealed a growing neovascular network at the choroid level in the OD (**Fig. 7**), while the examination of the left eye was completely normal. The findings led the team to acknowledge the need for further treatment. 

The abnormal activation of the sympathetic system was proven to be responsible for significant impairment in the choroidal vascular network and the overlying RPE [**15**]. In our case, choriocapillaris abnormalities were identified using ocular coherence tomography angiography. Compared to an age-matched healthy volunteer, the patient presented areas of reduced vessel density (**Fig. 8 A-C**). As previously mentioned, the decreased OCT-A signal could be due to RPE changes that determine shadowing artifacts or real hypoperfused lesions [**16**]. The evidence for choroidal vasculopathy being involved in the development of CSRC have been coming to light more recently, but the exact mechanism behind them is not yet understood. 

When it came to a possible hereditary component of the pathology, the patient denied any similar ocular manifestation within his family members, but his younger brother decided to present at the ophthalmology clinic for a routine check-up. His best corrected visual acuity was 1 and the posterior pole examination revealed: pigmentary changes with a “salt and pepper”-like pattern of the periphery in the absence of serous retinal detachments. OCT revealed normal macular architecture, but the surprising discovery was the abnormal choriocapillaris pattern identified by OCT-A. Areas of decreased signal were seen surrounding the macula, which was lacking any lesions that could be responsible for a shadowing effect, thus adding to the vasculopathy theory. Even though at the moment of the initial examination the man did not present any relevant clinical manifestations, the changes found by OCT-A imaging called for a close monitorization of the case.

It has been proven that microperimetry data correlate with anatomic findings in central serous chorioretinopathy. It has also been proven that retinal sensitivity ameliorates after fluid remission [**15**]. Our patient performed microperimetry examination at a time when no serous retinal detachments could be visualized neither by posterior pole examination, nor by OCT and therefore the results were within normal limits. 

While facing a case of multifocal, recurrent serous chorioretinopathy, the diagnosis is not the only challenging aspect, but also finding the right treatment for the patient. When the pathology was first described as MARC Syndrome, the only known effective treatment was photocoagulation [**1**]. In the 21st century, the treatment options are numerous without the presence of a certified gold standard. Photodynamic therapy with Verteporfin was investigated as a possible option to reduce choriocapillaris’ permeability. Beneficial in the beginning, this form of therapy can lead to significant side effects such as: RPE atrophy, choroidal neovascularization or ischemia. Considering the important role high cortisol levels are playing in the pathogenesis of the disease, treatment with mineralocorticoid receptor antagonists such as Spironolactone and Eplerenone was also proposed. Other oral therapeutic options include: antioxidants, beta-blockers, carbonic anhydrase inhibitors, Rifampicin and Methotrexate, but due to the small sample size, their benefit could not be certified [**17**-**19**]. Intravitreal injections with anti-VEGF factor have been analyzed as a possible treatment option in people with chronic CSRC for their role in reducing choroidal hyperpermeability by suppressing nitric oxide production [**2**,**3**,**20**,**21**]. The current approach recommends the use of intraocular injections for treating clearly identified CNV [**3**]. 

In our case, due to diffuse, recurrent, multifocal alteration of the retina and the lack of typical leakage spots on AFG, laser therapy was avoided and intravitreal injections with anti-VEGF agents were performed. Up to the moment of publication of the article, the patient received 4 intraocular injections in his left eye and 2 in his right one with episodes of reduced fluid accumulation. The patient’s benefits from intraocular treatment could be of greater significance now that choroid neovascularization was identified. 

## Conclusion

The case presents a rare variant of the classic CSRC affecting a middle-aged man without exposure to high levels of cortisol prior to the symptoms’ onset. The patient presents type A personality traits and moderate chronic stress exposure without a significant life-changing event preceding initial ocular manifestations. The prognosis is less favorable compared to a patient with the classical form of the disease who presents spontaneous recovery. The most likely evolution for our patient consists of recurrent episodes of fluid accumulation in the macula therefore affecting the visual acuity with spontaneous and under anti-VEGF agents’ reduction or shifting of the serous detachment areas.

The article also underlines the importance of choriocapillaris’s evaluation for the relatives of a patient with CSRC adding to the hypothesis that underlying choroidal vasculopathy could in fact lead to the changes seen in the disease. A hereditary factor could be present, but more in-depth studies are necessary to evaluate this theory. 

**Conflict of Interest statement**

The authors state no conflict of interest.

**Informed Consent and Human and Animal Rights statement**

Informed consent has been obtained from all individuals included in this study.

**Authorization for the use of human subjects**

Ethical approval: The research related to human use complies with all the relevant national regulations, institutional policies, is in accordance with the tenets of the Helsinki Declaration, and has been approved by the review board of “Iuliu Hațieganu” University of Medicine and Pharmacy Cluj-Napoca, Cluj, Romania.

**Acknowledgements**

None.

**Sources of Funding**

None.

**Disclosures**

None.
